# Endoplasmic reticulum stress‐dependent autophagy inhibits glycated high‐density lipoprotein‐induced macrophage apoptosis by inhibiting CHOP pathway

**DOI:** 10.1111/jcmm.14203

**Published:** 2019-02-12

**Authors:** Hua Tian, Yanyan Li, Panpan Kang, Zhichao Wang, Feng Yue, Peng Jiao, Nana Yang, Shucun Qin, Shutong Yao

**Affiliations:** ^1^ Key Laboratory of Atherosclerosis in Universities of Shandong and Institute of Atherosclerosis Taishan Medical University Taian China; ^2^ Affiliated hospital of Chengde Medical University Chengde Medical University Chengde China; ^3^ College of Nursing Taishan Medical University Taian China; ^4^ Department of Endocrinology Central Hospital of Taian Taian China; ^5^ College of Basic Medical Sciences Taishan Medical University Taian China

**Keywords:** apoptosis, autophagy, C/EBP homologous protein, endoplasmic reticulum stress, glycated high‐density lipoprotein, macrophage

## Abstract

This study was designed to explore the inductive effect of glycated high‐density lipoprotein (gly‐HDL) on endoplasmic reticulum (ER) stress‐C/EBP homologous protein (CHOP)‐mediated macrophage apoptosis and its relationship with autophagy. Our results showed that gly‐HDL caused macrophage apoptosis with concomitant activation of ER stress pathway, including nuclear translocation of activating transcription factor 6, phosphorylation of protein kinase‐like ER kinase (PERK) and eukaryotic translation initiation factor 2α, and CHOP up‐regulation, which were inhibited by 4‐phenylbutyric acid (PBA, an ER stress inhibitor) and the gene silencing of PERK and CHOP. Similar data were obtained from macrophages treated by HDL isolated from diabetic patients. Gly‐HDL induced macrophage autophagy as assessed by up‐regulation of beclin‐1, autophagy‐related gene 5 and microtubule‐associated protein one light chain 3‐II, which were depressed by PBA and PERK siRNA. Gly‐HDL‐induced apoptosis, PERK phosphorylation and CHOP up‐regulation were suppressed by rapamycin (an autophagy inducer), whereas aggravated by 3‐methyladenine (an autophagy inhibitor) and beclin‐1 siRNA. Administration of diabetic apoE^−/−^ mice with rapamycin attenuated MOMA‐2 and CHOP up‐regulation and apoptosis in atherosclerotic lesions. These data indicate that gly‐HDL may induce macrophage apoptosis through activating ER stress‐CHOP pathway and ER stress mediates gly‐HDL‐induced autophagy, which in turn protects macrophages against apoptosis by alleviating CHOP pathway.

## 1 INTRODUCTION

Exacerbated atherosclerosis is the most common and serious complication in patients with diabetes mellitus (DM), which is closely associated with hyperglycaemia and dyslipoproteinaemia, although the pathogenic mechanisms are incompletely elucidated. Hyperglycaemia may accelerate atherosclerotic progression by increasing the atherogenic potency of vascular smooth muscle cells 1 and triggering inflammation and oxidative stress.^2^ Another consideration is the non‐enzymatic formation of advanced glycation end products (AGEs) through long‐term exposure of proteins and lipids to glucose. AGEs have been imparted in the development and worsening of atherosclerosis by enhancing oxidized low‐density lipoprotein (ox‐LDL) formation, mediating inflammation and impairing reverse cholesterol transport.^3^, ^4^ The plasma level of high‐density lipoprotein (HDL) is inversely related to the occurrence of atherosclerotic cardiovascular diseases because of its protective potential, such as reverse cholesterol transport, anti‐inflammation and antioxidation. However, HDL in hyperglycaemic individuals is more highly glycated than in normoglycaemic individuals and the glycation of HDL impairs its antiatherogenic functions.^5^, ^6^ Moreover, glycated HDL (gly‐HDL) was demonstrated to induce endothelial apoptosis through mitochondrial dysfunction.^7^ Therefore, HDL exposure to hyperglycaemic conditions could contribute to the acceleration of atherosclerosis in DM patients. However, whether gly‐HDL could induce macrophage apoptosis and the potential precise mechanisms remain poorly understood.

Endoplasmic reticulum (ER) stress‐mediated apoptotic pathway has been generally recognized as an important mechanism for cell apoptosis and C/EBP homologous protein (CHOP), one of the specific proapoptotic molecules under ER stress, has been implicated in the progression of many diseases including DM and atherosclerosis. Pancreatic β cell dysfunction and/or apoptosis contribute to both type 1 and type 2 diabetes and are associated with ER stress. High‐glucose, free fatty acids and inflammatory challenge stimulate the ER stress‐CHOP pathway and oxidative stress in β cells.^8^ Conversely, CHOP deletion protects β cells against dysfunction and apoptosis in diet‐ and genetic‐induced diabetes models in mice.^9^ Macrophage apoptosis plays a key role in each stage of atherosclerosis, especially in the destabilization and rupture of atherosclerotic plaques, which may contribute to the majority of acute cardiovascular disease events.^10^ Accumulating evidence has shown that ER stress‐CHOP pathway‐mediated apoptosis in macrophages contributes to the instability of atherosclerotic plaques.^11^, ^12^ Previous studies including our own have demonstrated that ox‐LDL can initiate macrophage and endothelial cell apoptosis by elevating the CHOP pathway.^13^, ^14^, ^15^ Furthermore, our recent study has shown that CHOP mediates oxidized HDL‐induced macrophage apoptosis.^16^ Thus, we hypothesized that gly‐HDL may cause macrophage apoptosis by activating ER stress‐CHOP signals.

Additionally, ER stress has also been recognized as a mechanism for the stimulation of autophagy, a cellular degradation process responsible for the quality control and maintenance of energetic balance by the turnover of aggregated proteins and damaged subcellular organelles in lysosomes.^17^ Recently, multiple lines of evidence have revealed that both ER stress and autophagy are implicated in the pathogenesis of atherosclerotic plaque vulnerability and subsequent plaque rupture.^12^, ^18^, ^19^ However, the interaction of ER stress and autophagy in the progression of atherosclerosis induced by hyperglycaemia is currently unknown. In the present study, we investigated the roles of ER stress and autophagy in gly‐HDL‐induced macrophage apoptosis and the crosstalk between them in atherosclerosis.

## 2 MATERIALS AND METHODS

### 2.1 Reagents

Tunicamycin (TM), 4‐phenylbutyric acid (PBA), rapamycin, 3‐methyladenine (3‐MA), chloroquine (CQ), streptozotocin (STZ), oil red O, 3‐(4,5‐dimethylthiazol‐2‐y‐l)‐2,5‐ diphenyl‐2H‐tetrazolium bromide (MTT) and antibody against β‐actin were obtained from Sigma‐Aldrich (St Louis, MO, USA). Carboxymethyl lysine (CML) ELISA kits and Annexin V‐FITC apoptosis detection kits were obtained from BlueGene Biotech (Shanghai, China) and KeyGEN Biotech (Nanjing, China), respectively. Terminal deoxynucleotidyl transferase‐mediated dUTP nick end‐labelling (TUNEL) assay kit (In Situ Cell Death Detection kit, TMR red) and lactate dehydrogenase (LDH) assay kits were from Roche (Mannheim, Germany) and Jiancheng Biotech (Nanjing, China), respectively. Rabbit antibodies against activating transcription factor 6 (ATF6), phospho‐double‐stranded RNA‐activated protein kinase‐like ER kinase (p‐PERK) and phospho‐eukaryotic translation initiation factor 2α (p‐eIF2α) were obtained from Santa Cruz Biotechnology (Santa Cruz, CA, USA). Rabbit antibodies against glucose‐regulated protein 78 (GRP78), CHOP, P62 and rat antibody against monocyte plus macrophage (MOMA‐2) were purchased from Abcam (Cambridge, MA, USA). Rabbit antibodies against PERK, beclin‐1, autophagy‐related gene 5 (ATG5) and microtubule‐associated protein 1 light chain 3 (LC3) were obtained from Cell Signalling Technology (Danvers, MA, USA). Alexa Fluor 594‐labelled donkey anti‐rat and Alexa Fluor 488‐labelled donkey anti‐rabbit antibodies were from Molecular Probes (Eugene, OR, USA).

### 2.2 Subjects

Patients suffering from type 2 DM, defined by fasting glucose ≥7.0 mmol/L, were enrolled from the Department of Endocrinology, Central Hospital of Taian, Taian, China. The exclusion criteria included acute myocardial infarction, unstable angina or stroke within 6 months preceding the study, impaired renal or hepatic function, acute and chronic inflammatory diseases, thyroid diseases or other endocrine diseases. Healthy volunteers with fasting glucose <6.1 mmol/L were recruited as controls. Fasting plasma was collected for lipoprotein preparation and the measurement of blood glucose and lipid levels, which are shown in Table S1. The protocol was approved by the Ethics Committee of Taishan Medical University and all study subjects provided written informed consent.

### 2.3 Preparation of gly‐HDL

HDL (1.063–1.210 g/ml) was isolated from fresh fasting plasma of DM patients or healthy donors by density‐gradient ultracentrifugation as previously described.^16^ The separated HDL was dialysed at 4°C in endotoxin‐free phosphate buffered saline (PBS, pH = 7.4) in the presence of 1 mmol/L EDTA, sterilized with 0.22 μm filter, and stored in sealed tubes overlaid with nitrogen in the dark at 4°C. Gly‐HDL was prepared according to previous reports.^20^, ^21^ Briefly, native HDL (2 mg/ml) from healthy donors was incubated with 50 mmol/L glucose in PBS containing 2 mmol/L EDTA with nitrogen in the dark under sterile conditions at 37°C for 7 days. Glycation modification was prevented by dialysing extensively in PBS (pH 7.4) containing 1 mmol/L EDTA to remove free glucose. Additional samples of native HDL (n‐HDL, 2 mg/ml) were incubated identically, except for the exposure to glucose, to be used as a control. The extent of glycation in HDL was determined by measuring the abundance of CML by ELISA. The CML content in n‐HDL was 55.5 ± 10.8 pg/mg protein, whereas the CML levels in gly‐HDL and HDL from DM patients (DM‐HDL) were 197.9 ± 56.9 and 167.2 ± 57.6 pg/mg protein, respectively. The levels of endotoxin in HDL preparations were monitored by using the Limulus Amoebocyte Lysate kit (Bio Whittaker, Walkersville, MD) and no detectable amount (<50 pg/mg protein) of endotoxin was found in the HDL preparations used in the present study.

### 2.4 Cell culture and transfection of siRNA into cells

RAW264.7 macrophages (Type Culture Collection of the Chinese Academy of Sciences, Shanghai, China) were grown in Dulbecco's modified Eagle's medium (DMEM) containing 10% foetal bovine serum (FBS), 100 U/ml penicillin and 100 μg/ml streptomycin under a humidified condition of 5% CO_2_ at 37°C.

The transient transfection of small interfering RNA (siRNA) using Lipofectamine 2000 transfection reagent (Invitrogen, Carlsbad, CA) was performed according to the manufacturer's protocol. In brief, the siRNAs specific for PERK, CHOP and beclin‐1 (200 pmol) or control siRNA oligomers (200 pmol) were dissolved in 250 μL of Opti‐MEM I reduced serum medium (Invitrogen), and then gently mixed with 5 μL of Lipofectamine 2000 (Invitrogen) pre‐diluted in 250 μL of Opti‐MEM. After 20 min incubation at room temperature, the transfection complexes were added to cells grown in 6‐well plates in a final volume of 2.5 ml of medium and incubated for 6 h. Thereafter, the transfection medium was replaced with normal medium. After transfection for 48 h, the cells were exposed to gly‐HDL (100 mg/L) for 24 h. PERK siRNA (5′‐GUAAUGGUGCACUUUCAAUdTdT‐3′), CHOP siRNA (5′‐CUCUCCAGAUUC CAGUCAGdTdT‐3′) and beclin‐1 siRNA (5′‐CAAGUUCAUGCUGACCAAUdTdT‐3′) were obtained from Sigma‐Aldrich. The efficiency of siRNA‐silenced genes was validated by Western blot analysis.

### 2.5 Generation of type 2 diabetic atherosclerosis mouse model and rapamycin administration

Type 2 diabetic atherosclerosis mice were generated according to previous reports.^22^ Briefly, 5‐week‐old male apolipoprotein E knockout (apoE^−/−^) mice were obtained from Huafukang Bio‐technology Company (Beijing, China) and maintained on a high‐fat diet (20% fat, 20% sugar and 1.25% cholesterol) for 6 weeks. Thereafter, insulin‐resistant mice confirmed by an intraperitoneal glucose tolerance test were injected once with 75 mg/kg STZ intraperitoneally to induce partial insulin deficiency. Two weeks after STZ injection, mice with random blood glucose >11.1 mmol/L at least once in three separate tests were identified as type 2 diabetic mice, and then randomly distributed to receive intraperitoneal injection with either vehicle (DM group, n = 8) or rapamycin at the dose of 6 mg/kg per day (rapamycin group, n = 8) for additional 8 weeks. Eight male C57BL/6 mice fed a normal chow diet and injected intraperitoneally with 0.1% Tween‐80 were used as a control group. At the end of the experiment, the mice were killed by an overdose of pentobarbital and the hearts were perfused with ice‐cold PBS, removed transversely and fixed in 4% paraformaldehyde. The hearts with aortic roots were then embedded in optimal cutting temperature (OCT) compound and frozen at −80°C. The aortic roots were serially sectioned at 6 μm and the sections were collected on glass slides for oil red O staining, TUNEL and immunofluorescence analysis. All animal experiments followed the national guidelines for the care and use of animals and approved by the Laboratory Animals’ Ethical Committee of Taishan Medical University.

### 2.6 Intracellular lipid measurement

To observe lipid droplets in macrophages, the treated cells grown on cover glass were fixed with 4% paraformaldehyde for 20 min, washed with PBS, stained with oil red O (0.5%) in isopropanol for 30 min, and counterstained with haematoxylin for 5 min. The cells were observed using an Olympus BX51 microscope (Olympus, Tokyo, Japan), and then the lipid droplet content was analysed with Image‐Pro Plus 6.0 image analysis software (Media Cybernetics, LP, USA) and expressed as the average value of the integrated optical density (IOD) per cell.

Nile red staining was also used to determine intracellular lipid accumulation. The treated cells were washed with PBS and incubated with 1 μg/ml Nile red at room temperature for 30 min. After washing with PBS, the cells were resuspended in PBS and analysed using a FACScan flow cytometer (Becton Dickinson, San Jose, CA, USA) between 568 and 590 nm, and the results were expressed as the mean fluorescence intensity.

Atherosclerotic lesions in the mouse aortic root were analysed using oil red O staining as described in our previous report.^23^ Atherosclerotic lesions were observed by a microscope (Olympus) and the total mean lesion area was quantified from five sections per animal using Image‐Pro Plus software.

### 2.7 Cell viability and LDH assay

The viability of the treated cells was determined using MTT assay as previously reported 23 and expressed as the percentage of the optical density (OD) value of the treated cells relative to that of the untreated control cells.

LDH activity in media was measured using an assay kit according to the manufacturer's instructions to further assess the extent of cell injury.

### 2.8 Analysis of apoptotic cells

Cell apoptosis was determined using the Annexin V‐FITC/propidium iodide (PI) double‐staining assay and TUNEL assay, respectively, as described in our previous work.^16^ Briefly, after treatment, the cells were harvested, washed with PBS, and then incubated in 500 μL binding buffer containing 5 μL Annexin V‐FITC and 5 μL PI at room temperature in the dark for 15 min. The analysis of apoptosis percentage was performed using a FACScan flow cytometer (Becton Dickinson, San Jose, CA, USA).

For the TUNEL assay, the treated cells on coverslips or mouse aortic root cryosections were washed with PBS, fixed with 4% paraformaldehyde at room temperature in dark for 30 min, and then permeabilized with 0.1% Triton X‐100 for 3 min on ice. After washing with PBS, cells were incubated with TUNEL reaction mixture in a dark and humidified atmosphere at 37°C for 60 min and 4’, 6‐diamidino‐2‐phenylindole (DAPI) for 5 min, respectively, and then captured as digital images using fluorescence microscopy (Olympus BX51). Cell apoptosis was expressed as the percentage of the number of TUNEL‐positive cells to total cells or to the total atherosclerotic lesion area.

### 2.9 Immunofluorescence assay

Cells grown on coverslips were incubated with the corresponding intervention, and then washed with PBS, fixed with 4% paraformaldehyde for 20 min, permeabilized with 0.1% Triton‐X 100 for 5 min, and blocked with 10% donkey serum. Following incubation with ATF6 antibody (1:200 dilution) or LC3 antibody (1:200 dilution) overnight at 4°C, the cell coverslips were exposed to Alexa Fluor 488‐conjugated secondary antibody for 1 h at room temperature. After counterstaining with DAPI, the cell slips were mounted with antifade reagent and photographed using a laser scanning confocal microscope (Bio‐Rad Radiance 2100). Autophagosomal puncta accumulation labelled by LC3 was quantified using Image‐Pro Plus software, and expressed as the number of autophagosomal puncta per cell.

For immunofluorescence staining of mouse atherosclerotic lesions, serial aortic root cryosections were blocked with 10% donkey serum and incubated with the first primary antibodies specific for CHOP antibody (1:200 dilution), P62 (1:250 dilution) and MOMA‐2 antibody (1:400 dilution) overnight at 4°C. Then, the slides were exposed to a mixture of Alexa Fluor 594‐labelled donkey anti‐rat and Alexa Fluor 488‐labelled donkey anti‐rabbit secondary antibodies for 1 h. After counterstaining with DAPI, images were acquired under a fluorescence microscope (Olympus). The mean fluorescence intensity in the atherosclerotic lesion area was measured for each corresponding target protein using Image‐Pro Plus software.

### 2.10 Western blot

Western blotting was performed as previously reported.^14^, ^16^ Total proteins and nuclear proteins of the treated cells were extracted using radio immunoprecipitation assay buffer and nuclear extraction kits, respectively. Equal quantities of protein (30–60 μg) were separated by sodium dodecyl sulphate (SDS)‐polyacrylamide gel electrophoresis and then transferred to polyvinylidene fluoride membranes. The membranes were blocked with 5% BSA for 2 h at room temperature, and then incubated with primary antibodies overnight at 4°C, followed by incubation with horseradish peroxidase‐conjugated secondary antibodies for 2 h at room temperature. The immunoproteins were visualized using an enhanced chemiluminescence substrate system, and the protein blot intensities were quantified by Image‐Pro Plus software and normalized to house‐keeping protein (β‐actin or Histone) levels.

### 2.11 Quantitative real‐time PCR

RNA was extracted from the treated cells using the Trizol reagent (Invitrogen), and double‐stranded cDNA was synthesized using the Quantscript 1st strand cDNA synthesis kit (Tiangen Biological Chemistry, Beijing, China), according to the manufacturer's instructions. Quantitative real‐time PCR was performed using SYBR‐green PCR Realmaster mix kits (Tiangen Biological Chemistry) in a Rotor‐Gene Q real‐time PCR cycler (Qiagen, Shanghai, China), analysed by the Rotor‐Gene Q software (version 1.7, Qiagen), and then the mRNA amplification products were calculated on the basis of the relative expression method with the formula 2^–△△Ct^ as described previously.^16,23^ The primers used in this study were obtained from Sangon Biotech (Shanghai, China) and the sequences were as follows: 5’‐CCACCACACCTGAAAGCAGAA‐3’(forward primer) and 5’‐GGTGCCCCCAATTTCATCT‐3’(reverse primer) for CHOP, 5’‐ACATGGACCTGTTCCGCTCTA‐3’ (forward primer) and 5’‐TGGCTCCTTGCCATTGAAGA‐3’ (reverse primer) for GRP78, 5’‐CGGGGACCTGACTGACTACC‐3’ (forward primer) and 5’‐AGGAAGGCTGGAAGAGTGC‐3’ (reverse primer) for β‐actin.

### 2.12 Statistical analysis

Results were expressed as mean ± SD. Multiple comparisons were analysed by one‐way analysis of variance with Student‐Newmann‐Keuls test and comparison between two groups was determined by Student's *t* test using the SPSS13.0 software for Windows. *P *<* *0.05 were considered statistically significant.

## 3 RESULTS

### 3.1 Gly‐HDL induces lipid accumulation and apoptosis in RAW264.7 cells

**Figure 1:**
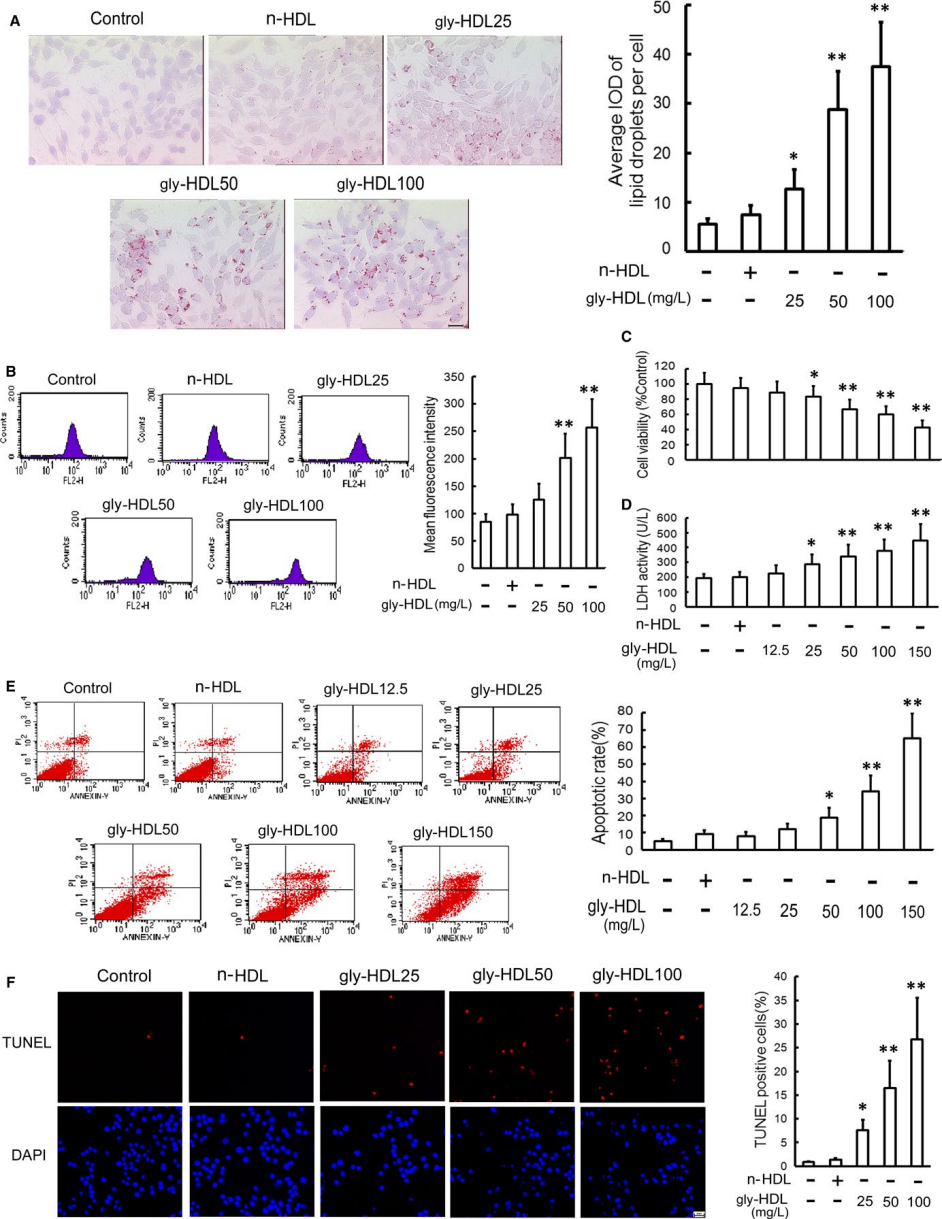
Gly‐HDL attenuates cell viability and induces apoptosis in RAW264.7 cells. RAW264.7 cells were exposed to the indicated doses of gly‐HDL and n‐HDL (100 mg/L) for 24 h. The levels of intracellular neutral lipid and cholesteryl ester were measured using oil red O staining; (A) and Nile red staining; (B) respectively. Scale bar = 20 μm. Cell viability; (C) and LDH activity in media; (D) were measured by MTT assay and a kit, respectively. E, Cell apoptosis was determined by flow cytometry, and the total apoptotic cells were shown on the right side of the panel (Annexin *v* staining alone or together with PI). F, Cell apoptosis was measured by TUNEL assay and represented by the percentage of TUNEL‐positive cells to the total cells. Scale bar = 20 μm. Data are expressed as the mean ± SD of at least four independent experiments. *P <* *0.05, **P <* *0.01 vs control group

Because the apoptosis of lipid‐overloaded macrophage‐derived foam cells plays a key role in the progression and rupture of atherosclerotic plaques,^10^, ^11^, ^12^ we examined whether gly‐HDL could induce lipid accumulation and apoptosis in macrophages. Both oil red O staining (Figure 1A) and Nile red staining (Figure 1B) showed that treatment with gly‐HDL, but not n‐HDL, for 24 h significantly increased the lipid content in RAW264.7 cells in a concentration‐dependent manner. As seen in Figure 1C and D, exposure of RAW264.7 cells to gly‐HDL dose‐dependently decreased cell viability and resulted in LDH leakage. Moreover, both Annexin V‐FITC/PI double staining and TUNEL assay revealed that gly‐HDL led to a significant increase in apoptosis (Figure 1E and F).

### 3.2 ER stress‐CHOP pathway mediates macrophage apoptosis induced by gly‐HDL

**Figure 2:**
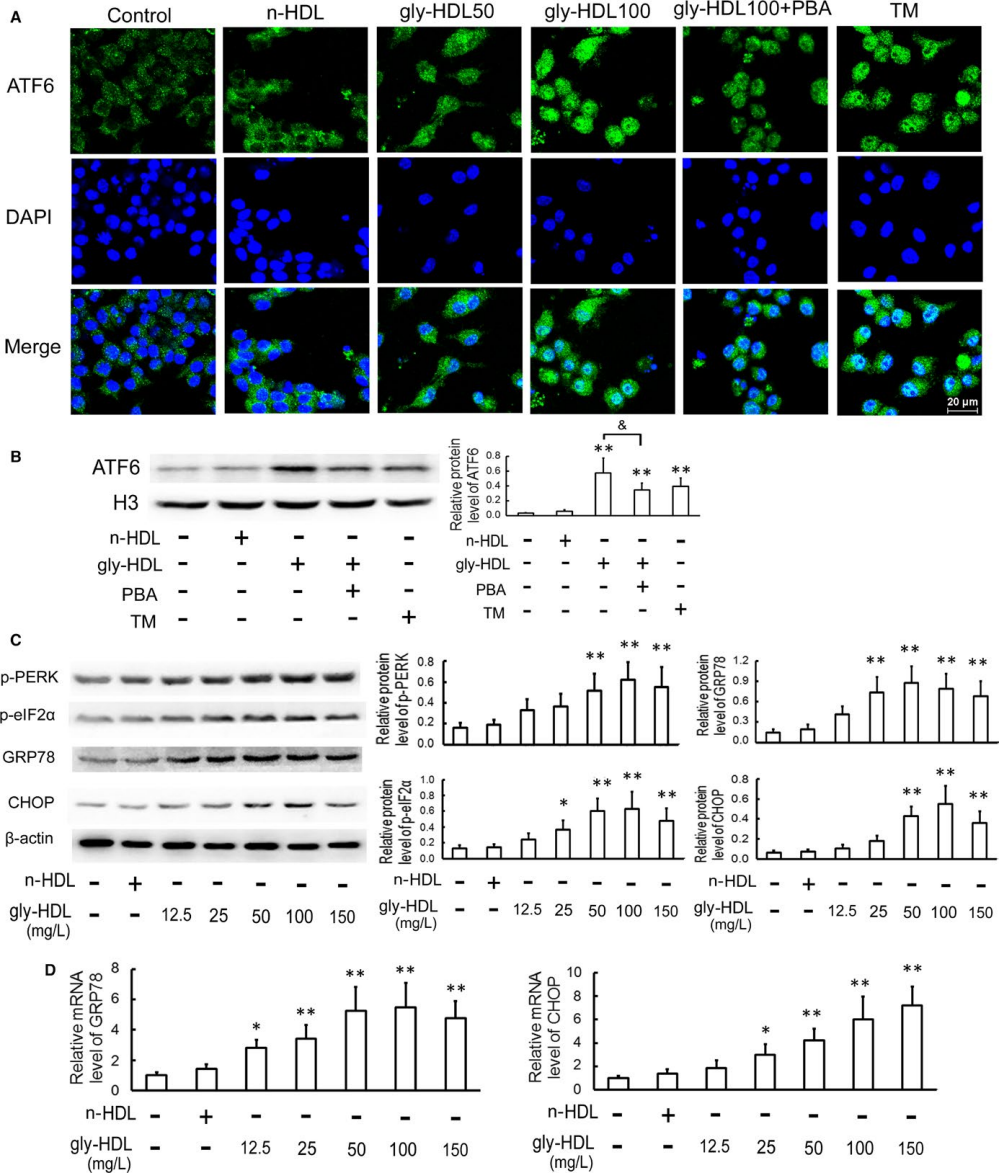
Gly‐HDL activates ER stress‐CHOP pathway in RAW264.7 cells. A, Cells were pre‐incubated with or without 5 mmol/L of PBA for 1 h, and then exposed to gly‐HDL (50 or 100 mg/L) or TM (4 mg/L) for 24 h. Immunofluorescence experiments showed ATF6 labelled by Alexa Fluor 488 (green) and nuclei stained with DAPI (blue). Representative fluorescent images captured by a laser scanning confocal microscope are shown. Scale bar = 20 μm. B, Cells were pre‐treated with or without 5 mmol/L of PBA for 1 h, and then exposed to gly‐HDL (100 mg/L) or TM (4 mg/L) for 24 h. The protein level of ATF6 in nuclear extracts was analysed by Western blotting and normalized to Histone (H3) level. C and D, Cells were treated as described in Figure 1 E, and then the protein and mRNA levels of ER stress markers were analysed by Western blotting and quantitative real‐time PCR, respectively. Data are expressed as the mean ± SD of at least three independent experiments. *P <* *0.05, **P <* *0.01 vs control group; *P *< 0.05

**Figure 3:**
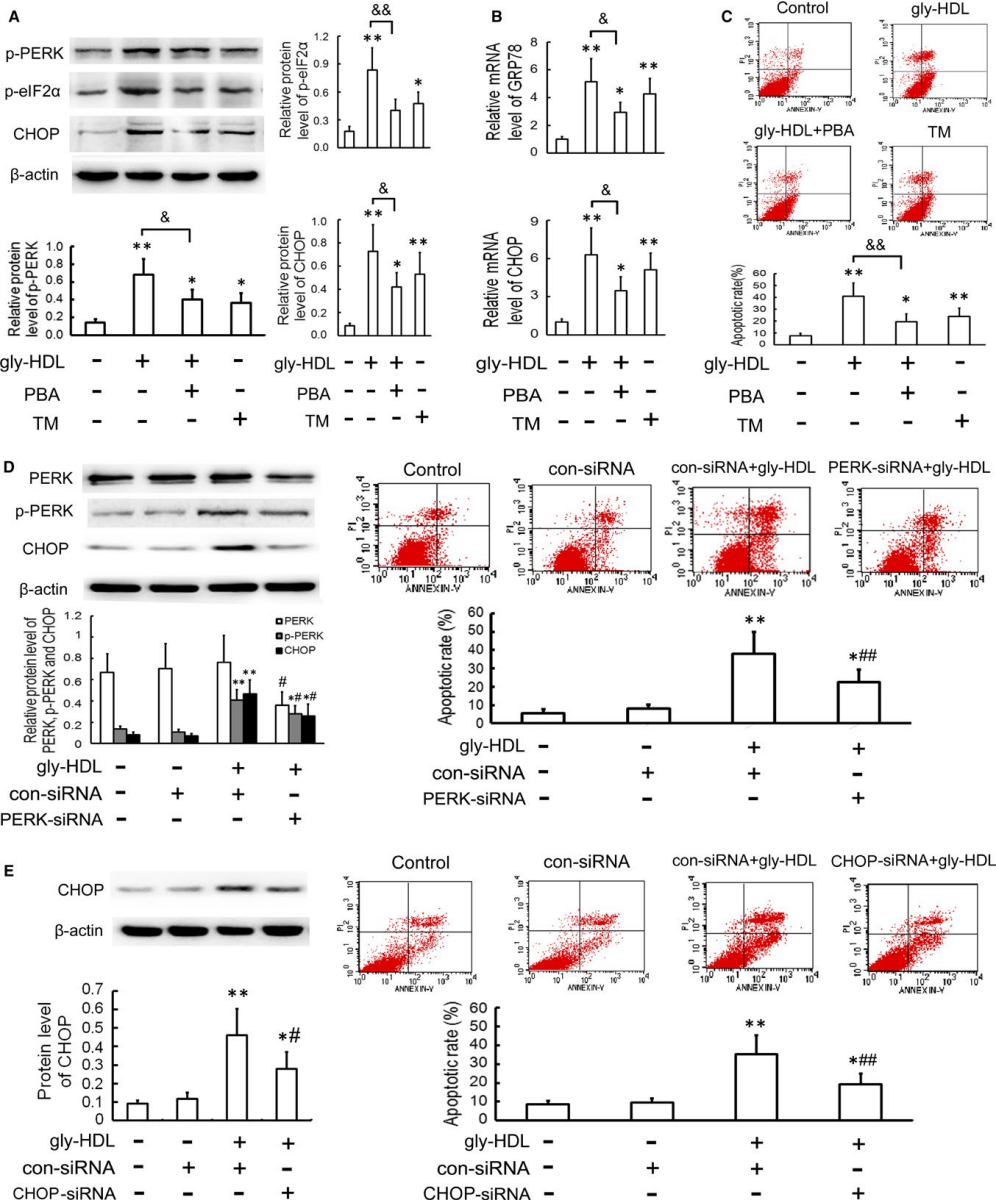
Attenuation of ER stress‐CHOP pathway inhibits gly‐HDL‐induced macrophage apoptosis. A and B, RAW264.7 cells were exposed to 100 mg/L gly‐HDL or TM (4 mg/L) in the presence or absence of PBA (5 mmol/L) for 24 h, and then the protein and mRNA levels of ER stress markers were measured by Western blotting and quantitative real‐time PCR, respectively. C, Cell apoptosis was determined by flow cytometry and the total apoptotic cells were shown on the right side of the panel (Annexin V staining alone or together with PI). D and E, RAW264.7 cells were transfected with siRNA specific for PERK or CHOP, treated with 100 mg/L gly‐HDL for 24 h, and then PERK, p‐PERK and CHOP protein levels and cell apoptosis were analysed by Western blotting and flow cytometry, respectively. Data are expressed as the mean ± SD of at least three independent experiments. *P <* *0.05, **P <* *0.01 vs control group; ^&^ *P *<* *0.05, ^&&^ *P *<* *0.01; ^#^ *P *<* *0.05, ^##^ *P *<* *0.01 vs gly‐HDL group transfected with con‐siRNA

ER stress‐CHOP pathway has been demonstrated to play a key role in macrophage apoptosis,^11^, ^12^, ^14^, ^16^ so we evaluated the effect of gly‐HDL on CHOP and its two important upstream molecules ATF6 and PERK. As indicated in Figure 2 and Figure 3A–C, similar to TM (an ER stress inducer), gly‐HDL, but not n‐HDL, significantly elevated the detection of ER stress markers including nuclear translocation of ATF6, phosphorylation of PERK and eIF2α coupled with the increased expression of GRP78 and CHOP both at the protein and mRNA levels. However, PBA, an ER stress inhibitor, markedly depressed gly‐HDL‐induced ER stress‐CHOP pathway activation and cell apoptosis.

To further identify whether ER stress‐CHOP pathway is implicated in gly‐HDL‐induced macrophage apoptosis, we determined whether genetic inhibition of PERK and CHOP could inhibit gly‐HDL‐induced apoptosis. As shown in Figure 3D and E, transfection of PERK or CHOP‐specific siRNA exhibited significant attenuation of gly‐HDL‐induced CHOP up‐regulation and cell apoptosis.

Furthermore, mouse peritoneal macrophages were also used in this study. As shown in Figure S1, gly‐HDL caused injury of mouse peritoneal macrophages as determined by the decreased cell viability and the increased LDH leakage and apoptosis, which were inhibited by PBA. Additionally, PBA inhibited gly‐HDL‐induced CHOP up‐regulation at both the protein and mRNA levels.

### 3.3 HDL from diabetic patients induces intracellular lipid accumulation and CHOP‐mediated macrophage apoptosis

**Figure 4:**
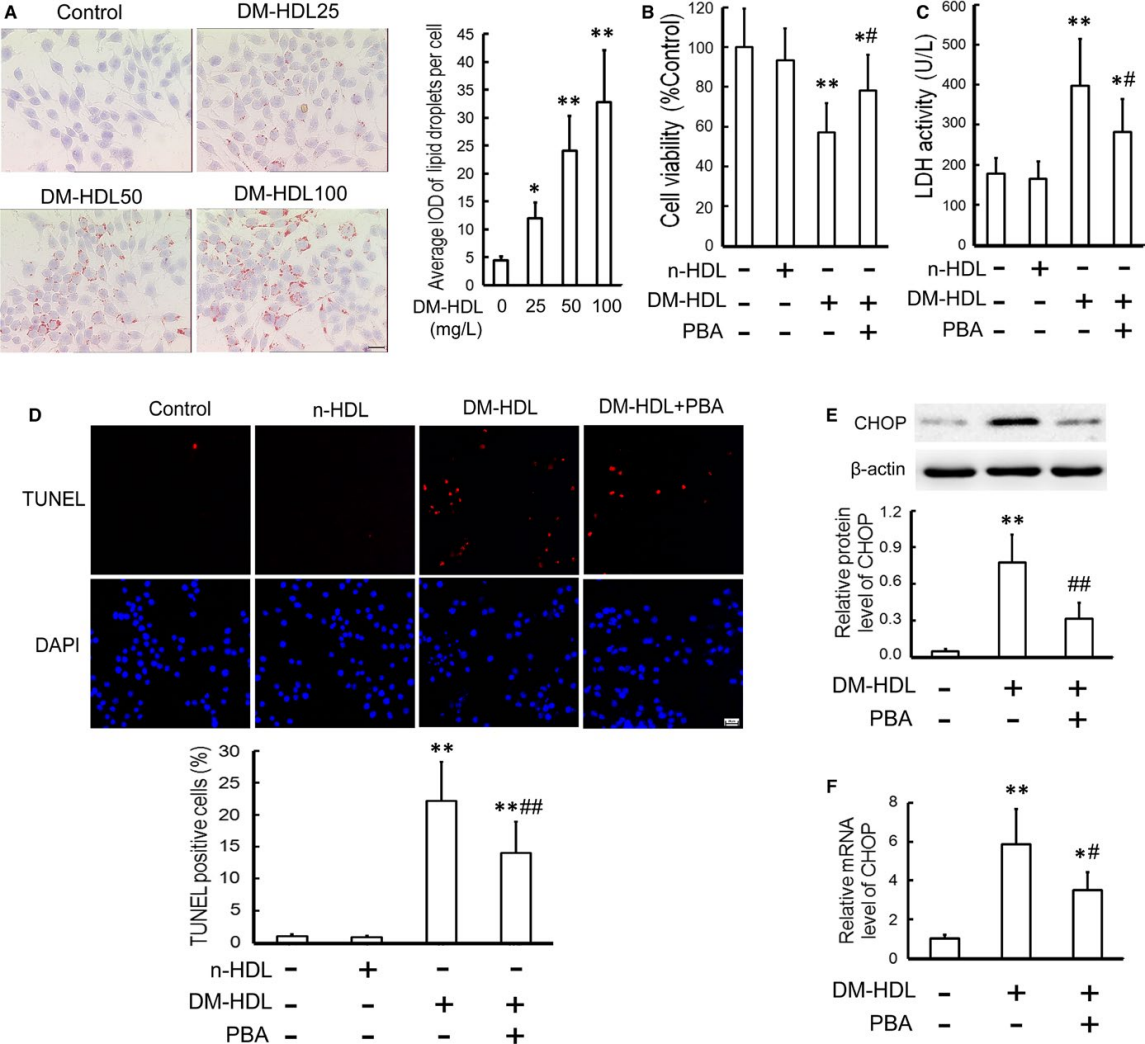
HDL from DM patients (DM‐HDL) induces lipid accumulation and CHOP‐mediated macrophage apoptosis. (A) RAW264.7 cells were exposed to DM‐HDL (25, 50 and 100 mg/L) for 24 h, and then the intracellular lipid level was measured using oil red O staining. Scale bar = 20 μm. RAW264.7 cells were pre‐incubated with or without 5 mmol/L PBA for 1 h, and then exposed to DM‐HDL (100 mg/L) or n‐HDL (100 mg/L) for 24 h. Cell viability; (B) and LDH activity in the media; (C) were measured by MTT assay and a kit, respectively. D, Cell apoptosis was analysed by TUNEL assay. Scale bar =20 μm. E and F, CHOP protein and mRNA levels were determined by Western blotting and quantitative real‐time PCR, respectively. Data are expressed as the mean ± SD of at least three independent experiments. *P <* *0.05, **P <* *0.01 vs control group; ^#^ *P *<* *0.05, ^##^ *P *<* *0.01 vs DM‐HDL group

To obtain further evidence for the pathophysiological significance of gly‐HDL in clinical diseases, we isolated HDL from DM patients (DM‐HDL) and determined its effect on intracellular lipid level and CHOP‐mediated apoptosis in RAW264.7 macrophages. The CML content in DM‐HDL was 167.2 ± 57.6 pg/mg protein, which is much higher than that in n‐HDL (55.5 ± 10.8 pg/mg protein, *P *<* *0.01). As shown in Figure 4A–D, exposure to DM‐HDL resulted in intracellular lipid accumulation and macrophage injury as determined by the attenuated cell viability and increased LDH leakage and apoptosis, which were decreased by PBA. Additionally, DM‐HDL increased the protein and mRNA expression of CHOP, which were suppressed by PBA (Figure 4E and F).

### 3.4 Gly‐HDL triggers autophagy through ER stress in RAW264.7 cells

**Figure 5:**
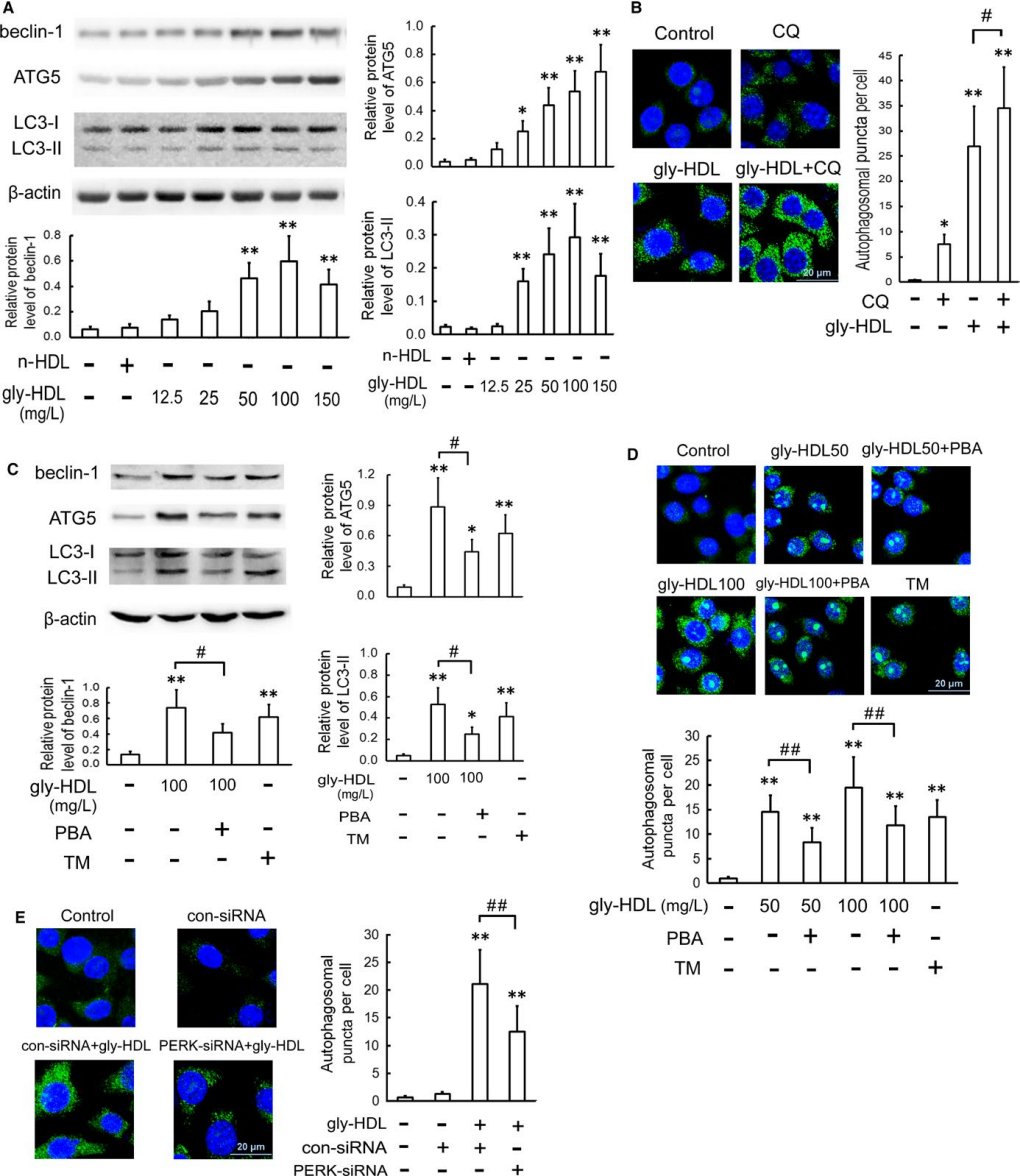
Gly‐HDL triggers autophagy through ER stress in RAW264.7 cells. A, RAW264.7 cells were exposed to the indicated doses of gly‐HDL and n‐HDL (100 mg/L) for 24 h, and then the protein levels of autophagy markers were analysed by Western blotting. B, Cells were treated with 100 mg/L gly‐HDL in the presence or absence of CQ (5 μmol/L) for 24 h. Immunofluorescence experiments showed LC3 labelled by Alexa Fluor 488 (green) and nuclei visualized by DAPI (blue). Representative fluorescent images were photographed by a laser scanning confocal microscope and autophagosomal puncta/cells were quantified. C and D, Cells were exposed to 50 or 100 mg/L gly‐HDL or TM (4 mg/L) in the presence or absence of PBA (5 mmol/L) for 24 h, and then the protein levels of autophagy markers and LC3 puncta were determined by Western blotting and immunofluorescence experiments, respectively. E, Cells were transfected with PERK siRNA, treated with 100 mg/L gly‐HDL for 24 h, and then LC3 puncta were determined by immunofluorescence experiments. Scale bar = 20 μm. Data are expressed as the mean ± SD of at least three independent experiments. *P <* *0.05, **P <* *0.01 vs control group; ^#^ *P *<* *0.05, ^##^ *P *<* *0.01

To verify whether gly‐HDL induces autophagy in macrophages, we detected the changes in autophagy markers in gly‐HDL‐treated RAW264.7 cells. As shown in Figure 5A, gly‐HDL, but not n‐HDL, elevated the levels of autophagy marker proteins such as beclin‐1, ATG5 and LC3‐II (an indicator of autophagosome formation) with increasing concentration. To further confirm the effect of gly‐HDL on autophagosome formation, immunofluorescence analysis of the specific autophagosomal membrane marker LC3 in RAW264.7 cells was performed, because the number of LC3 puncta per cell is usually an accurate measure of autophagosomes. Compared with the control cells, the gly‐HDL‐treated cells exhibited a greater number of autophagosomal puncta (Figure 5B).

Since LC3‐II formation is transient during the activation of autophagy and the protein can be rapidly degraded in lysosomes, the elevated LC3‐II might also be indicative of attenuated lysosomal degradation^24^. Thus, to more accurately determine autophagic flux, RAW264.7 cells were exposed to gly‐HDL in the presence or absence of CQ, a lysosomal inhibitor that inhibits lysosomal degradation of LC3‐II. As shown in Figure 5B, incubation of cells with CQ increased LC3 puncta. The addition of CQ heightened a further increase in LC3 puncta than that in the cells treated with gly‐HDL or CQ alone. These results suggest that gly‐HDL promotes autophagosome formation rather than disrupts its maturation into the autophagolysosome.

It has been reported that the activation of ER stress pathway is a cellular process triggering autophagy response.^25^ To determine whether ER stress mediates the stimulatory effect of gly‐HDL on autophagy, we detected gly‐HDL‐induced autophagy after treating the cells with PBA, an ER stress inhibitor. As shown in Figure 5C and D, PBA pre‐treatment significantly decreased gly‐HDL‐enhanced autophagy as evidenced by decreased protein levels of beclin‐1, ATG5 and LC3‐II as well as attenuated autophagosomal puncta, whereas TM, an ER stress inducer, triggered autophagy. Moreover, compared with the gly‐HDL‐treated group, PERK siRNA decreased the number of autophagosomal puncta (Figure 5E), indicating that gly‐HDL‐induced autophagy is ER stress‐dependent.

### 3.5 Autophagy attenuates cell apoptosis by inhibiting CHOP up‐regulation in gly‐HDL‐treated RAW264.7 cells

**Figure 6:**
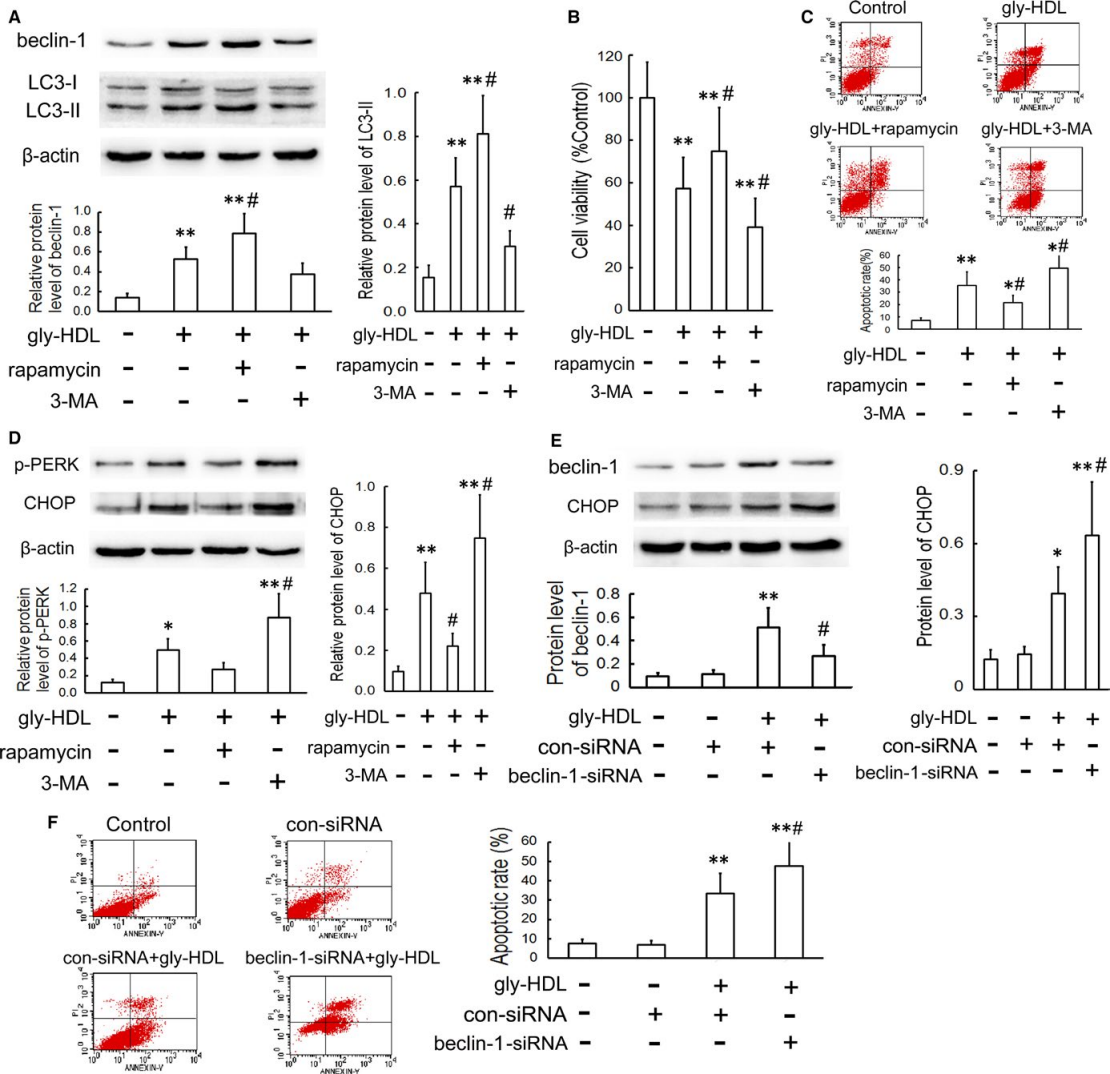
Autophagy attenuates cell apoptosis by inhibiting CHOP up‐regulation in gly‐HDL‐treated RAW264.7 cells. RAW264.7 cells were exposed to gly‐HDL (100 mg/L) in the presence or absence of rapamycin (1 μmol/L) or 3‐MA (3 mmol/L) for 24 h. (A) The protein levels of beclin‐1 and LC3 were analysed by Western blotting. Cell viability; (B) and apoptosis (C) were measured by MTT assay and flow cytometry, respectively. D, The protein levels of p‐PERK and CHOP were analysed by Western blotting. Cells were transfected with beclin‐1 siRNA, treated with 100 mg/L gly‐HDL for 24 h, and then beclin‐1 and CHOP levels; (E) and cell apoptosis; (F) were measured by Western blotting and flow cytometry, respectively. Data are expressed as the mean ± SD of at least three independent experiments. *P <* *0.05, **P <* *0.01 vs control group; ^#^ *P *<* *0.05 vs gly‐HDL group

Previous studies have revealed that up‐regulated autophagy in macrophages and vascular endothelial cells can exert protective functions against cell death and attenuate atherosclerosis.^26^, ^27^, ^28^ To elucidate the role of autophagy in gly‐HDL‐induced macrophage apoptosis and its mechanism, we investigated the effect of gly‐HDL on cell apoptosis after regulating autophagic activity. Pre‐treatment with rapamycin, an autophagy inducer, enhanced a further increase in the levels of beclin‐1 and LC3‐II induced by gly‐HDL, whereas pre‐incubation with 3‐MA, an autophagy inhibitor, suppressed gly‐HDL‐up‐regulated beclin‐1 and LC3‐II levels (Figure 6A). Additionally, rapamycin alleviated gly‐HDL‐enhanced cell injury as assessed by elevated cell viability and decreased apoptosis. In contrast, 3‐MA aggravated gly‐HDL‐enhanced cell injury (Figure 6B and C).

It has been reported that bidirectional regulation exists between autophagy and ER stress.^29^ Thus, we explored whether autophagy mitigates gly‐HDL‐induced apoptosis by regulating the ER stress‐CHOP pathway. As shown in Figure 6D, pre‐incubation with rapamycin suppressed PERK phosphorylation and CHOP up‐regulation in cells treated with 100 mg/L gly‐HDL for 24 h. Conversely, 3‐MA aggravated gly‐HDL‐enhanced ER stress‐CHOP pathway.

To further confirm the inhibitory effect of autophagy on gly‐HDL‐induced apoptosis via the CHOP pathway in macrophages, RAW264.7 cells were transfected with beclin‐1 siRNA prior to exposure to gly‐HDL. As shown in Figure 6E and F, beclin‐1 siRNA exaggerated gly‐HDL‐enhanced CHOP up‐regulation and macrophage apoptosis. These data suggest that enhanced autophagy in RAW264.7 cells may mitigate gly‐HDL‐induced cell apoptosis by inhibiting ER stress‐CHOP pathway.

To further identify the relationship between ER stress‐CHOP pathway and autophagy, the time course of up‐regulation of p‐PERK, CHOP and beclin‐1 was analysed. As shown in Figure S2A, p‐PERK was significantly up‐regulated at an earlier time point than beclin‐1 in cells treated with 100 mg/L gly‐HDL, and CHOP expression increased significantly at the 16‐hour time point and reached a maximum at 24 hours, which appeared more later than that of p‐PERK and beclin‐1. In addition, at the 8 hours of treatment of cells with 100 mg/L gly‐HDL, pre‐treatment with rapamycin and 3‐MA had no significant effect on p‐PERK level, whereas PERK siRNA significantly inhibited gly‐HDL‐induced beclin‐1 up‐regulation (Figure S2B and C). These data above suggest that ER stress may mediate gly‐HDL‐induced autophagy at an earlier stage, whereas autophagy in turn protects macrophages against apoptosis by inhibiting excessive activation of CHOP pathway at the late stage.

### 3.6 Enhanced autophagy by rapamycin inhibits CHOP up‐regulation, apoptosis and atherosclerotic lesions in type 2 diabetic apoE^−/−^ mice

**Figure 7:**
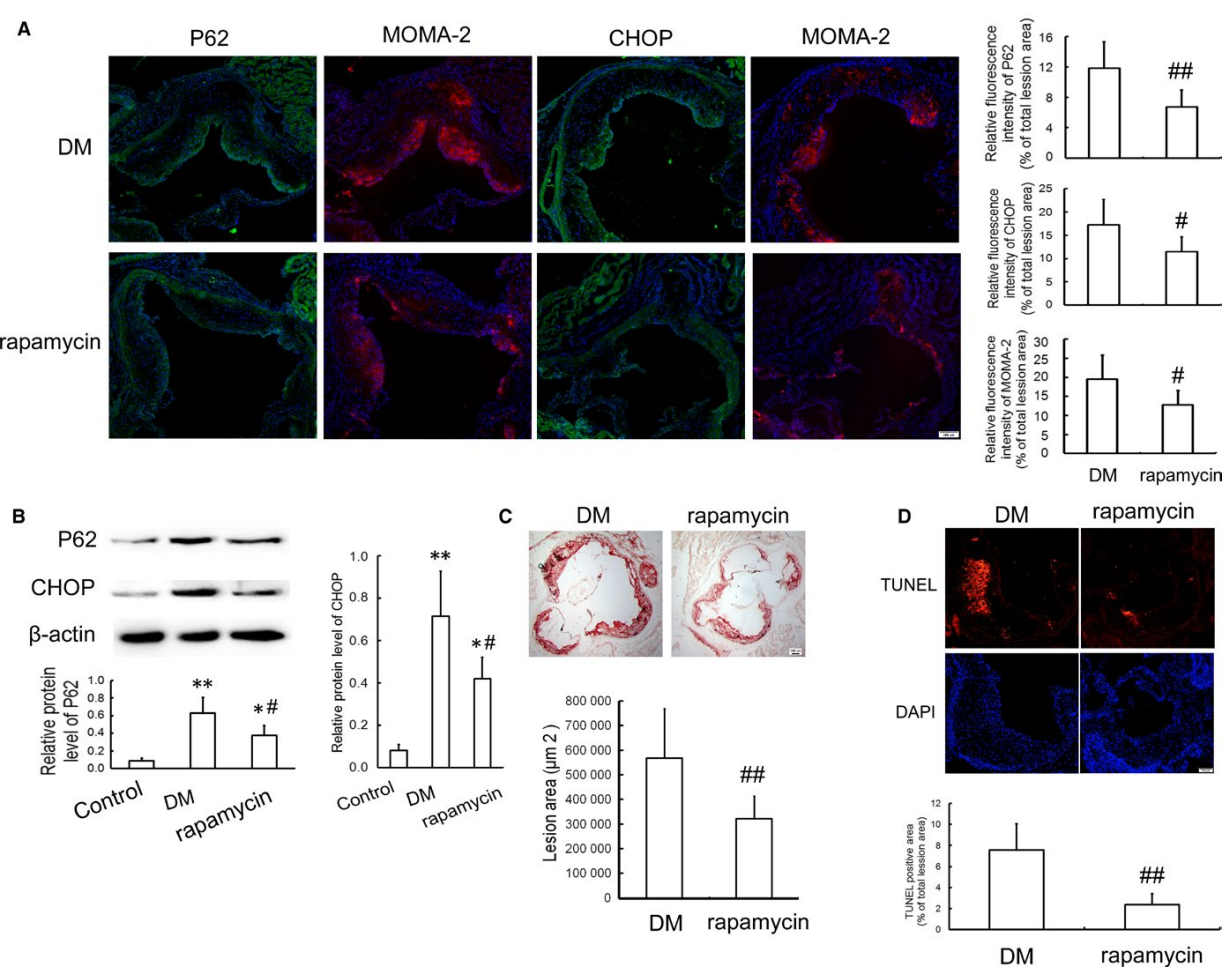
Effects of rapamycin on CHOP expression and apoptosis in atherosclerotic lesions of diabetic apoE^−/−^ mice. Type 2 diabetic atherosclerosis apoE^−/−^ mice were generated and given vehicle (DM group) or 6 mg/kg of rapamycin (rapamycin group) per day by intraperitoneal injection for 8 weeks. Male C57BL/6J mice were fed normal chow diet as a control group. A, Immunofluorescent staining showing the expression of P62, CHOP and MOMA‐2. Scale bar =100 μm. The relative fluorescence intensity for P62, CHOP (green) and the macrophage‐dense areas (MOMA‐2, red) in the lesions was calculated. B, Western blot analysis of P62 and CHOP in the aortic arch. C, Atherosclerotic lesions stained by oil red O. Scale bar=100 μm. D, Cell apoptosis in atherosclerotic lesions determined by TUNEL staining. Red, TUNEL‐positive cell; blue, nuclei stained by DAPI. Scale bar =100 μm. Data are presented as the mean ± SD of at least three independent experiments. *P <* *0.05, **P <* *0.01 vs control group; ^#^ *P *<* *0.05, ^##^ *P *<* *0.01 vs DM group

To extend our in vitro findings, we established type 2 diabetic atherosclerosis model using apoE^−/−^ mice to explore the effect of rapamycin on CHOP expression, apoptosis and atherosclerotic lesions. As shown in Figure 7A and B, treatment with rapamycin for 8 weeks reduced the protein level of P62, an autophagic substrate, indicating that autophagy was enhanced. Additionally, the number of MOMA‐2‐positive macrophages and the level of CHOP in atherosclerotic lesions from rapamycin‐administered mice were much lower than those from DM mice. Furthermore, plaque area and cell apoptosis in the aortic roots from DM mice were remarkably reduced by rapamycin treatment (Figure 7C and D).

## 4 DISCUSSION

Macrophage apoptosis is a key event in the formation and rupture of atherosclerotic plaques and accelerated atherosclerosis is the major complication of DM, a metabolic disease affecting people worldwide. Accumulating evidence indicates that AGEs play a crucial role in the atherosclerotic process that accompanies diabetes. As one of AGEs, gly‐HDL may also be involved in accelerated atherogenesis, but its role in macrophage apoptosis and the underlying precise mechanisms remain poorly understood. The present study was the first to report that gly‐HDL elicited macrophage apoptosis by activating the ER stress‐CHOP pathway and the up‐regulation of autophagy was a cellular protective response that attenuated ER stress‐CHOP‐mediated macrophage apoptosis induced by gly‐HDL, which was supported by the following findings. First, gly‐HDL induced intracellular lipid accumulation, macrophage injury and apoptosis with concomitant activation of ER stress pathway, including nuclear translocation of ATF6, phosphorylation of PERK and eIF2α coupled with up‐regulation of GRP78 and CHOP. Second, gly‐HDL‐induced apoptosis and CHOP up‐regulation were suppressed by PBA (an ER stress inhibitor) and the gene silencing of PERK and CHOP. Third, HDL isolated from diabetic patients induced intracellular lipid accumulation, macrophage injury, apoptosis and CHOP up‐regulation, which were inhibited by PBA. Fourth, gly‐HDL induced macrophage autophagy as assessed by the up‐regulation of beclin‐1, ATG5 and LC3‐II as well as autophagosome formation, which were decreased by PBA and PERK siRNA. Fifth, gly‐HDL‐induced macrophage injury and apoptosis, PERK phosphorylation and CHOP up‐regulation were suppressed by rapamycin (an autophagy inducer), but aggravated by 3‐MA (an autophagy inhibitor) and beclin‐1 siRNA. Sixth, the administration of type 2 diabetic apoE^−/−^ mice with rapamycin attenuated the up‐regulation of MOMA‐2 and CHOP, and ameliorated cell apoptosis in atherosclerotic lesions.

It is generally accepted that HDL exhibits antiatherosclerotic potentials attributing to its functions such as promoting reverse cholesterol transport, preventing LDL oxidation and protecting endothelial cells. Thus, raising HDL level was regarded as a promising approach to ameliorate atherosclerosis development. Cholesteryl ester transfer protein (CETP) inhibitors are thought to be applicable for significantly elevating HDL levels in animal models.^30^ However, the excess mortality and no apparent effects to decrease carotid intima‐media thickness and reduce the risk of recurrent cardiovascular events using CETP inhibitors such as dalcetrapib and torcetrapib 31, 32 have led to a reconsideration of which is more important for the atheroprotective properties of HDL, quantity or quality.^33^ Therefore, the impact of HDL modification on HDL abilities and pathological significance has drawn much attention from researchers. Previous studies have revealed that oxidative modification of HDL impairs its antiatherogenic abilities from many aspects such as attenuating paraoxonase activity 34 and inhibiting reverse cholesterol transport.^35^ Furthermore, oxidized HDL may become pro‐atherogenic by promoting ROS production, increasing proinflammatory factor expression,^36^ inducing the proliferation and migration of vascular smooth muscle cells,^37^ and causing apoptosis in endothelial cells 38 and macrophages.^16^ In addition to oxidative modification, HDL can also be glycated in diabetes and the biological properties of HDL are altered including the reduced paraoxonase activity and anti‐inflammatory function, and the decreased capacity to mediate reverse cholesterol transport.^5^, ^6^, ^39^ Moreover, gly‐HDL has been shown to induce endothelial apoptosis,^7^ and cause cellular senescence and foam cell formation.^40^ The results in this study showed that gly‐HDL led to intracellular lipid accumulation, as well as macrophage injury in a dose‐dependent manner as determined by attenuated cell viability and increased LDH leakage and apoptosis.

ER stress activation in macrophages is frequently observed in atherosclerotic lesions and contributes to the development of early and advanced atherosclerotic lesions.^41^ Multiple lines of evidence including our previous data have revealed that ER stress mediates macrophage‐derived foam cell formation by up‐regulating scavenger receptors including CD36 and lectin‐like oxidized LDL receptor‐1.^42^, ^43^ However, persistent or uncontrolled ER stress can activate apoptotic cascades and then affect the vulnerability of plaques to rupture.^12^ The activation of the ER stress‐CHOP pathway has been confirmed to contribute to advanced‐lesional macrophage apoptosis and plaque rupture, whereas reducing CHOP expression can inhibit macrophage apoptosis and decrease plaque vulnerability within advanced lesional atherosclerosis.^11^, ^12^, ^13^, ^44^ Our previous data have revealed that ox‐LDL and ox‐HDL initiate CHOP‐induced macrophage apoptosis by triggering PERK and ATF6 pathways, whereas D4F, an apolipoprotein A‐I mimetic peptide, suppresses ox‐LDL‐induced macrophage apoptosis by attenuating the ER stress‐CHOP pathway.^14^, ^16^, ^23^ It has been reported that glycation of paraoxonase 1 by high glucose instigates ER stress to induce endothelial dysfunction, and glucolipotoxicity induces THP‐1 monocyte apoptosis by up‐regulating CHOP expression.^45^, ^46^ Our present experiments showed that gly‐HDL induced ER stress, as demonstrated by the elevated nuclear translocation of ATF6, phosphorylation of PERK and eIF2α as well as up‐regulation of GRP78 and CHOP, which was similar to the effects induced by TM. Conversely, gly‐HDL‐triggered apoptosis and activation of ER stress‐CHOP pathway were suppressed by PBA (an ER stress inhibitor) and the gene silencing of PERK and CHOP. Consistent with the results above, exposure to HDL isolated from diabetic patients caused intracellular lipid accumulation and induced macrophage injury and apoptosis as well as CHOP up‐regulation, which were suppressed by PBA. These results indicate that the ER stress‐CHOP pathway may play a key role in gly‐HDL‐induced macrophage apoptosis.

Autophagy is an essential catabolic process responsible for degrading and recycling cell constituents, such as misfolded proteins and damaged organelles, by transferring them into double‐membrane autophagosomes that subsequently fuse with lysosomes. There is evidence that an interaction exists between autophagy and ER stress in the regulation of cell apoptosis and the progression of atherosclerosis, neurodegenerative disorders and diabetic cardiomyopathy, whereas the precise role and potential mechanisms have not been fully clarified.^17^, ^47^, ^48^ ER stress has been identified as a mechanism for the activation of autophagy via PERK, inositol‐requiring enzyme 1‐dependent pathways.^25^ In the present study, we observed autophagy in gly‐HDL‐treated macrophages, as assessed by the up‐regulated beclin‐1, ATG5 and LC3‐II levels as well as autophagosome formation. Additionally, the administration of chloroquine enhanced a further increase in autophagosome formation than that in the cells treated with gly‐HDL or chloroquine alone, suggesting that gly‐HDL heightened autophagic flux. Furthermore, enhanced autophagy in cells treated with gly‐HDL for 24 h was inhibited by PBA (an ER stress inhibitor) and PERK siRNA, whereas TM (an ER stress inducer) triggered autophagy, indicating that ER stress may mediate gly‐HDL‐induced autophagy. To further identify the relationship between ER stress pathway and autophagy, the time course of up‐regulation of p‐PERK, CHOP and beclin‐1 were analysed. The data showed that p‐PERK was significantly up‐regulated at an earlier time point than beclin‐1 in cells treated with gly‐HDL, and CHOP up‐regulation reached a maximum at 24 hours, which appeared much later than that of p‐PERK and beclin‐1. In addition, at the 8 hours of treatment of cells with gly‐HDL, pre‐treatment with rapamycin (an autophagy inducer) and 3‐MA (an autophagy inhibitor) had no significant effect on p‐PERK level, whereas PERK siRNA significantly inhibited gly‐HDL‐induced beclin‐1 up‐regulation, suggesting that in addition to mediating ER stress, PERK also mediates gly‐HDL‐induced autophagy at an earlier stage.

Although some reports indicate that prolonged and severe stress can initiate the autophagy‐dependent cell death pathway and result in cell injury,^49^, ^50^ autophagy has been demonstrated to exhibit a pro‐survival function in response to a variety of stimuli, such as oxidative stress, metabolic stress and ER stress,^51^, ^52^, ^53^ and play a protective role in advanced atherosclerosis.^27^, ^54^ Additional studies have shown that 7‐ketocholesterol‐induced autophagy protected vascular smooth muscle cells against apoptosis by inhibiting ER stress 26 and AGE‐modified bovine serum albumin (AGE‐BSA)‐activated autophagy‐attenuated AGE‐BSA‐induced injury in human vascular endothelial cells.^28^ Conversely, inadequate autophagy contributes to endothelial dysfunction in patients with diabetes 55 and enhances vascular smooth muscle cell death and atherosclerosis,^56^ so autophagy may be a target for the therapy of cardiovascular metabolic disease.^57^ In our study, the induction of autophagy by rapamycin alleviated gly‐HDL‐enhanced ER stress‐CHOP pathway and cell apoptosis. In contrast, the attenuation of autophagy by 3‐MA and gene silencing of beclin‐1 aggravated the activation of ER stress‐CHOP pathway and apoptosis in macrophages exposed to gly‐HDL. These results indicate that elevated autophagy in response to gly‐HDL may be an adaptive pro‐survival mechanism that protects against excessive ER stress‐CHOP‐mediated macrophage apoptosis and atherosclerotic progression related to DM. This standpoint was further supported by our in vivo studies that showed that the induction of autophagy by rapamycin reduced the levels of MOMA‐2 and CHOP, and ameliorated atherosclerotic lesions and apoptosis in type 2 diabetic apoE^−/−^ mice.

Collectively, our study demonstrated that gly‐HDL induced lipid accumulation and macrophage apoptosis through activating ER stress‐CHOP pathway and that ER stress mediated gly‐HDL‐induced autophagy response at an earlier stage, which in turn protected macrophages against apoptosis by alleviating the excessive activation of ER stress‐CHOP pathway to some extent. These findings provide novel insights into the crucial direct role of gly‐HDL and the crosstalk between ER stress and autophagy in diabetes‐associated atherosclerotic pathogenesis, suggesting that enhanced autophagy is a beneficial adaptive response that alleviates the excessive activation of ER stress‐CHOP pathway and suppresses gly‐HDL‐induced macrophage apoptosis.

## AUTHOR CONTRIBUTION

HT performed the data collection and analysis, and drafted the manuscript. YL, PK and ZW performed cell culture, WB, oil red O staining and MTT assay. FY collected the blood samples of DM patients and healthy donors. PJ performed flow cytometry analysis and quantitative real‐time PCR analysis. NY, YL and ZW performed animal experiments. SQ and SY were responsible for the study design, funding and data analysis. All authors have read and approved the final manuscript.

## CONFLICT OF INTEREST

The authors declare no conflict of interest.

## Supporting information


** **
