# Supplementary material for: Endoplasmic reticulum stress‐dependent autophagy inhibits glycated high‐density lipoprotein‐induced macrophage apoptosis by inhibiting CHOP pathway
Source: J Cell Mol Med. 2019 Feb 12;23(4):2954–69. doi: 10.1111/jcmm.14203 (PMC6433656; doi:10.1111/jcmm.14203)
Supplement: Supplementary file 1 [file JCMM-23-2954-s001.pdf]

Endoplasmic reticulum stress-dependent autophagy inhibits glycated high-density lipoprotein-induced macrophage apoptosis by inhibiting CHOP pathway

Hua Tian[1, §], Yanyan Li[1, §], Panpan Kang[2], Zhichao Wang[3], Feng Yue[4], Peng Jiao[1], Nana Yang[1], Shucun Qin[1,*], Shutong Yao[1, 5, *]

Supplementary Information

Supplementary table 1. Clinical and biochemical characteristics of diabetes mellitus (DM) patients and healthy controls.

	Healthy controls (n = 35)	DM Patients (n = 47)	P
Age (years)	45.7 ± 9.7	59.7 ± 11.3	<0.01
Gender(male/female)	19/16	22/25	
Fasting glucose (mmol/L)	5.02 ± 0.53	10.27 ± 2.98	<0.01
Triglycerides (mmol/L)	0.99 ± 0.25	1.52 ± 0.65	<0.01
Total cholesterol (mmol/L)	4.55 ± 0.94	4.79 ± 0.95	0.25
HDL-cholesterol (mmol/L)	1.45 ± 0.35	1.23 ± 0.34	<0.01
LDL-cholesterol (mmol/L)	2.52 ± 0.55	2.95 ± 0.82	<0.01

Data are expressed as the mean ± SD. Differences between DM group and control group were tested using the Student's t test.

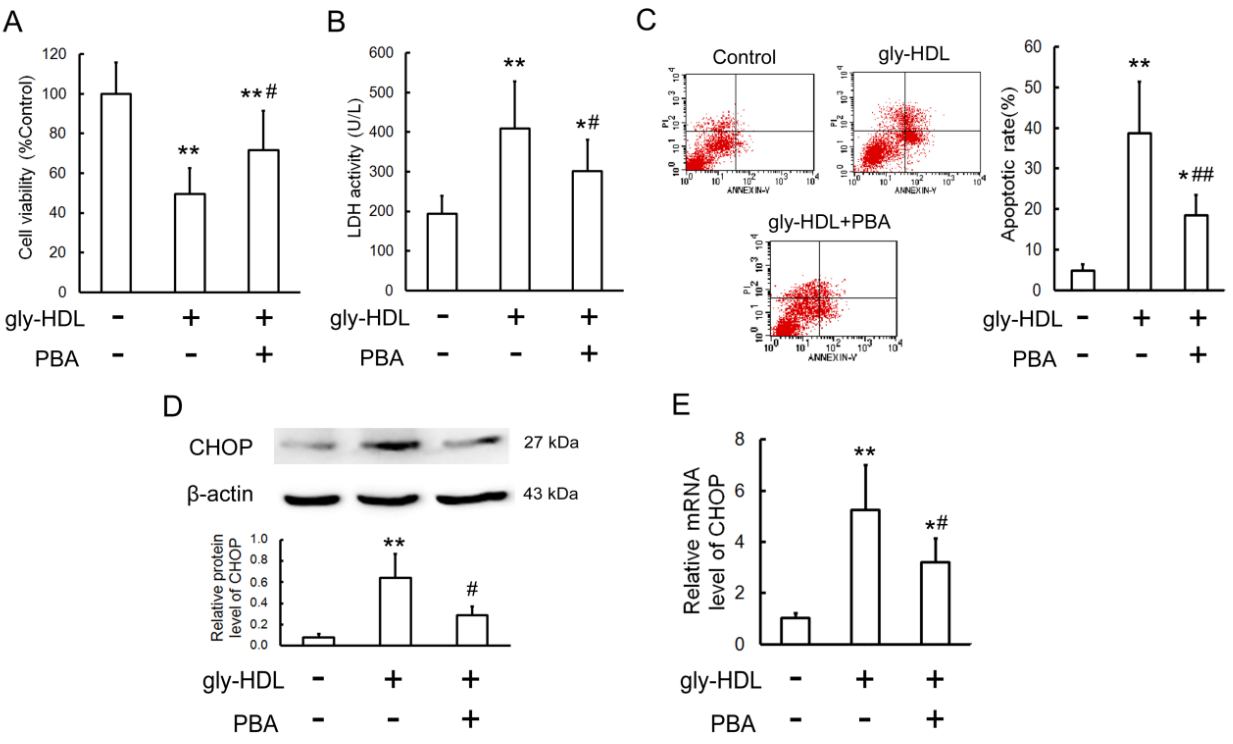

Supplementary figure S1. Gly-HDL induces CHOP-mediated apoptosis in mouse peritoneal macrophages. Peritoneal macrophages from C57BL/6J mice were harvested with PBS three days after intraperitoneal injection with 1 ml of 4% thioglycollate and cultured in DMEM containing 10% FBS. Cells were preincubated with or without PBA (5 mmol/L) for 1 h, and then exposed to 100 mg/L gly-HDL for 24 h. Cell viability (**A**) and LDH activity in media (**B**) were measured by MTT assay and a kit, respectively. (**C**) Cell apoptosis was determined by flow cytometry and the total apoptotic cells were expressed as the right side of the panel (Annexin V staining alone or together with PI). (**D** and **E**) The protein and mRNA levels of CHOP were analyzed by Western blot and quantitative real-time PCR, respectively. Data are expressed as the mean ± SD of at least three independent experiments. **P*<0.05, ***P*<0.01 versus control group; #*P*<0.05, ##*P*<0.01 versus gly-HDL group.

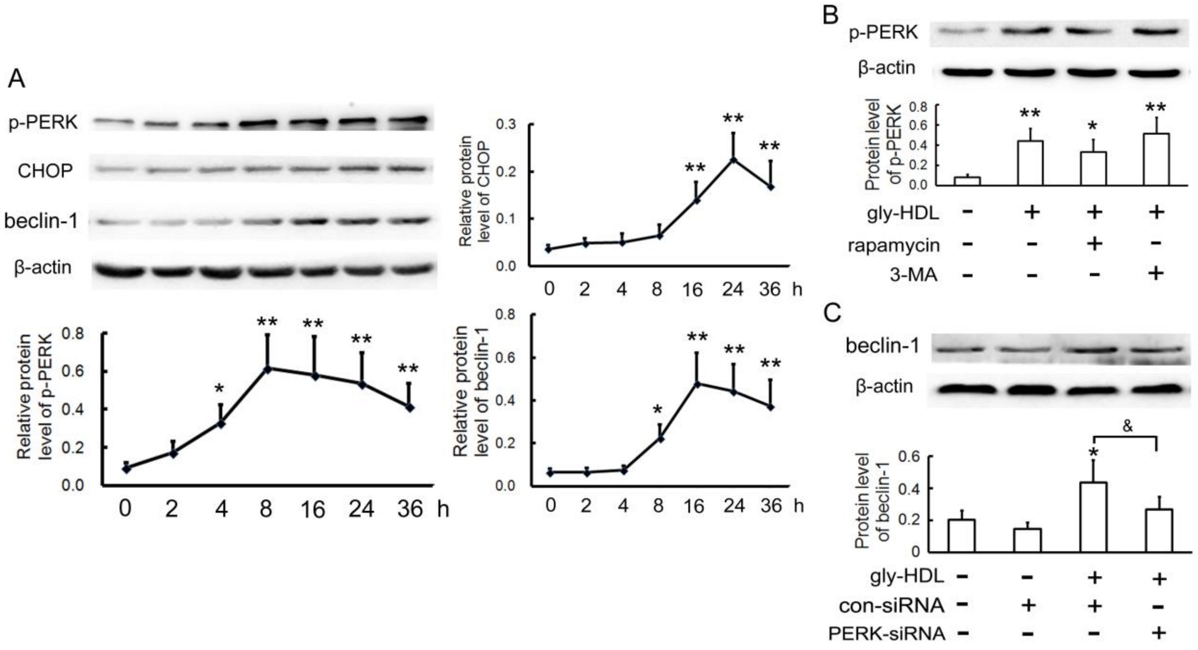

Supplementary figure S2. The changes of p-PERK, CHOP and beclin-1 in RAW264.7 cells caused by gly-HDL in different periods. (**A**) Cells were treated with 100 mg/L gly-HDL for the indicated periods. (**B**) Cells were exposed to gly-HDL (100 mg/L) in the presence or absence of rapamycin (1 μmol/L) or 3-MA (3 mmol/L) for 8 h. (**C**) Cells were transfected with PERK siRNA and treated with 100 mg/L gly-HDL for 8 h. The protein levels of p-PERK, CHOP and beclin-1 were analyzed by Western blotting. Data are expressed as the mean ± SD of three independent experiments. **P*<0.05, ***P*<0.01 *versus* control group; &*P*<0.05.
